# Skin-Related Properties and Constituents from the Aerial Parts Extract of *Persicaria senticosa*

**DOI:** 10.1155/2020/6627752

**Published:** 2020-12-19

**Authors:** Yun-Hyeok Choi, Jae Yeon Lee, Ji Eun Lee, Yeon Woo Jung, Wonsik Jeong, Seong Su Hong, Young-Rak Cho, Chun Whan Choi

**Affiliations:** ^1^Natural Products Research Team, Biocenter, Gyeonggido Business and Science Accelerator, Suwon 16229, Republic of Korea; ^2^Medicinal Evaluation Team, Biocenter, Gyeonggido Business and Science Accelerator, Suwon 16229, Republic of Korea

## Abstract

In the course of screening for cosmetic ingredients by measuring antioxidant and antiwrinkle and whitening and anti-inflammatory activities, skin-related activity was tested using 1,1-diphenyl-2-picrylhydrazyl (DPPH) radical scavenging, 2,2′-azino-bis(3-ethylbenzothiazoline-6-sulfonic acid) (ABTS) radical scavenging, elastase inhibition, tyrosinase inhibition, and nitric oxide assay. Several Polygonaseae extracts were found to show potent activity. The results showed that the *Persicaria senticosa* methanolic extract has the 1,1­diphenyl-2­picrylhydrazyl (DPPH) and ABTS radical scavenging activities (IC_50_ 61.0 and 17.5 *μ*g/mL). In the elastase inhibition assay and nitric oxide assay, the IC_50_ of methanolic extract of *Persicaria senticosa* was 739.7 *μ*g/mL and 71.8 *μ*g/mL. The *Persicaria senticosa* 70% ethanolic extract partitioned with n-hexane, CH_2_Cl_2_, EtOAc, n-BuOH, and aqueous fractions. The purification of EtOAc soluble layer was by column chromatography separation and MPLC analysis of Compounds **1-7**. It was identified as loliolide (**1**), quercetin-3-O-glucoside (**2**), quercetin-3-O-glucuronide (**3**), 4-methoxy caftraric acid (**4**), kaempferol-3-(6-methylglucuronide) (**5**), quercetin-3-(6-methylglucuronide) (**6**), and quercetin (**7**). Structure was elucidated by a combination of 1D and 2D NMR and MS spectrometry as well as comparison with reported literatures. Radical scavenging effect on DPPH, tyrosinase inhibition, and nitric oxide assay on several compounds from *Persicaria senticosa* was found to show potent activity. The results showed that Compound **7** has the NO assay (IC_50_29.7 *μ*M). For DPPH, the IC_50_ of Compounds **2**, **3**, **5**, and **7** was 39.6, 31.2, 37.0, and 22.7 *μ*M. In tyrosinase inhibitory activity, the IC_50_ of Compound **7** was 14.3 *μ*M.

## 1. Introduction

*Persicaria senticosa* is an annual plant in the family Polygonaseae, which is distributed in the whole of Korea. Since early times, this plant has been used as folk medicine with beneficial effects for the treatment of various diseases such as removing the swelling parts of the wound or carbuncles and cellulitis and circulating blood and removing blood stagnation. Recent studies have shown that *Persicaria senticosa* had anti-inflammatory effects [1]; however, this plant has not been reported as bioactive cosmetic ingredients. And previous phytochemical studies on the flowers, stem, and roots of the genus Polygonum have revealed that various flavonoids and phenolic compounds such as hydroxybenzoic acid, rutin, quercetin-3-O-glucuronide, quercetin-3-O-glucoside, and luteolin-7-O-rutinoside were considered the major active components [2, 3]. These active compounds exhibit diverse pharmacological effects, such as anti-inflammatory, antiulcer, antihypertensive, and anticancer effects [4]. During the screening for cosmetic ingredients by measuring the radical scavenging effect on 1,1-diphenyl-2-picrylhydrazyl (DPPH), ABTS radical scavenging, elastase inhibition, tyrosinase inhibition and nitric oxide assay, several Polygonum extracts were found to show potent activity.

## 2. Materials and Methods

### 2.1. Plant Materials and General Procedures of Natural Products

*Persicaria senticosa* was collected from Seo-myeon, Chuncheon-si, Gangwon-do, Korea, in 2016 (GPS: N 37° 55′ 30.1^″^, E 127° 37′ 57.0^″^, altitude: 434 m). Voucher specimens (G071) were authenticated by Dr. Chun Whan Choi. *Persicaria senticosa* were deposited at the herbarium of Biocenter, Gyeonggido Business & Science Accelerator, Suwon, South Korea (Fig. S3). The 99.9% methanol extract of thirty-four Polygonum was obtained from the Korea Plant Extract Bank at the Korea Research Institute of Bioscience and Biotechnology (Daejeon, Korea). ^1^H and ^13^C NMR experiments were performed on a Bruker Ascend 700 MHz spectrometer with tetramethylsilane (TMS). LC-ESI-MS was obtained on a Triple TOF 5600+ instrument (AB SCIX, USA) and HRESI-MS on a LTQ Orbitrap XL instrument (Thermo, USA). Thin-layer chromatography (TLC) was conducted on Silica gel 60 F_254_ (Merck, Germany) and Silica gel 60 RP-18 F_254S_ (Merck, Germany) plates. Column chromatography(CC) was performed using Silica gel 60 (70~230 mesh, Merck, Germany), ODS-A (12 nm S-7 *μ*m, YMC GEL, Japan), and preparative HPLC was performed on LC-8A (Shimadzu, Japan).

### 2.2. NO Assay

RAW 264.7 cells were seeded in 96-well plates (5 × 10^4^ cells/well) and were treated with sample for 1 h prior to LPS (1 *μ*g/mL) stimulation for 24 h. The negative control was treated with serum-free media. The amount of nitrite, a stable metabolite of NO, was measured by using Griess reagent (1% sulfanilamide and 0.1% naphthylethylenediamine dihydrochloride in 2.5% phosphoric acid). Absorbance was subsequently measured at 540 nm using an ELISA reader. The quantity of nitrite was determined from a standard curve for sodium nitrite [5–7].

### 2.3. Cell Cytotoxicity Assay

RAW 264.7 cells were plated at a density of 5 × 10^4^ cells/well in 96-well plates. Cells were treated with samples for 1 h prior to LPS (1 *μ*g/mL) stimulation for 24 h. MTT (5 mg/mL in PBS) was added to each well and incubated for 2 hr. The medium was removed from the wells by aspiration, DMSO was added to each well, and the plate was shaken. The absorbance of each well was measured at a wavelength of 540 nm using an ELISA reader. Data are presented as the mean ± standard deviation of three replicates.

### 2.4. DPPH Radical Scavenging Activity Assay

DPPH radical scavenging activity was measured by using the method described by Blois [8] and Ozgen et al. [9]. DPPH solution dissolved in methanol was added to the sample, which was diluted to the required concentration, and the reaction was carried out at room temperature for 30 min. Absorbance was measured at 517 nm using an ELISA reader. The antioxidant butylated hydroxyanisole (BHA) was used as a positive control, and the IC_50_ value of the sample was determined.

### 2.5. ABTS Radical Scavenging Activity

ABTS radical scavenging activity was measured by using a previously described method [10, 11]. ABTS+ was formed by mixing 7 mM ABTS solution and 2.45 mM potassium persulfate (K_2_S_2_O_8_) solution with ABTS: K_2_S_2_O_8_ (2 : 1 ratio) for 12–16 h to form a cation (ABTS^+^). The absorbance was measured at 734 nm using an ELISA reader. BHA, an antioxidant, was used as the positive control.

### 2.6. Tyrosinase Inhibition Assay

Tyrosinase inhibitory activity was measured by using the method described by Yagi et al. [12]. The reaction was carried out in 0.1 M potassium phosphate buffer (pH 6.5) containing 1.5 mM L-tyrosine and 1250 units/mL mushroom tyrosinase. The reaction mixture was incubated at 37°C for 20 min. The test samples were assayed for tyrosinase inhibition by measuring its effect on tyrosinase activity using SpectraMax 190PC microplate ELISA reader at 490 nm. Arbutin and kojic acid were used as a positive control, and the IC_50_ value of the sample was determined.

### 2.7. Elastase Inhibition Assay

The reaction was carried out in 0.5 mM Tris buffer (pH 8.5) containing 1 mg/mL N-succinyl-(Ala)_3_-p-nitroanilide and 0.6 unit/mL elastase. The reaction mixture was incubated at 25°C for 10 min. The test samples were assayed for elastase inhibition by measuring its effect on elastase activity using an ELISA reader at 405 nm. Ursolic acid was used as a positive control, and the IC_50_ value of the sample was determined [13].

### 2.8. Statistical Analysis

Statistical analysis of the data was performed by PRIZM5 software (GraphPad, CA, USA), and data are presented as mean ± SD. One-tailed Student's *t*-test was used for analyzing the significance of difference between groups, and *P* < 0.05 was considered statistically significant.

## 3. Results and Discussion

### 3.1. Skin-Related Properties of Plants Extracts in the Family Polygonaseae

Radical scavenging effect on DPPH, ABTS radical scavenging, elastase inhibition, tyrosinase inhibition, and nitric oxide assay on several Polygonum extracts were found to show potent activity. The results showed that the *Persicaria japonica* and *Rumex longifolius* has the NO assay (IC_50_ 45.3 and under 25.0 *μ*g/mL). For DPPH, the IC_50_ of methanolic extract of *Polygonum ciliinerve*, *Polygonum alpinum*, and *Persicaria chinensis* was 36.9, 15.4, and 19.2 *μ*g/mL, respectively. In the ABTS radical scavenging activity, the IC_50_ of methanolic extract of *Polygonum cuspidate* and *Polygonum alpinum* was 5.2 and 5.3 *μ*g/mL, respectively. In tyrosinase inhibitory activity, the IC_50_ of methanolic extract of *Polygonum sachalinens* root and *Polygonum cuspidata* was 289.0 and 483.9 *μ*g/mL, respectively. In the elastase inhibition assay, the IC_50_ of methanolic extract of *Polygonum sachalinense*, *Polygonum cuspidatum*, *Persicaria hydropiper*, *Persicaria sieboldi*, *Polygonum orientale*, *Persicaria lapathifolia*, *Persicaria dissitiflora*, *Rumex acetosella*, *Rumex crispus*, *Polygonum alpinum*, *Persicaria longiseta*, *Persicaria chinensis*, *Persicaria japonica*, *Persicaria viscofera*, *Persicaria conspicua*, *Rheum palmatum*, and *Persicaria lapathifolia* was under 100 *μ*g/mL (Table 1).

### 3.2. Skin-Related Properties of *Persicaria senticosa* Fractions

Radical scavenging effect on 1,1-diphenyl-2-picrylhydrazyl(DPPH), ABTS radical scavenging, elastase inhibition, and nitric oxide assay on several *Persicaria senticosa* fractions was found to show potent activity. The results showed that the CH_2_Cl_2_ and EtOAc fractions have the NO assay (under 25 and 44.64 *μ*g/mL, respectively). The DPPH and ABTS radical scavenging activities of EtOAc fractions were 13.7 and 5.0 *μ*g/mL. In the elastase inhibition assay, the IC_50_ of n-hexane and CH_2_Cl_2_ fractions was under 100 *μ*g/mL (Table 2).

### 3.3. Isolation and Determination of Compounds from *Persicaria senticosa* Extract

**Scheme 1:**
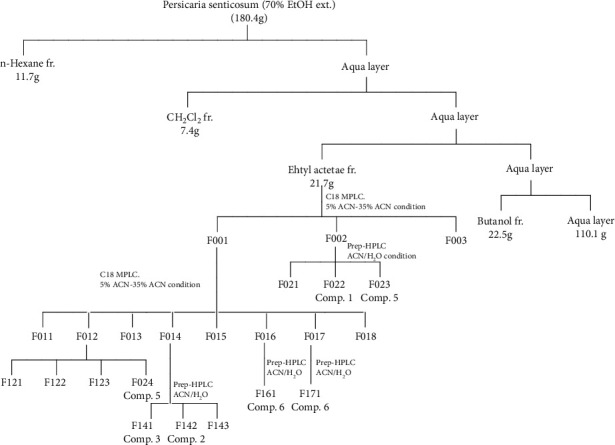
Isolation of the Compounds **1**–**7** from aerial parts of *Persicaria senticosa.*

**Figure 1:**
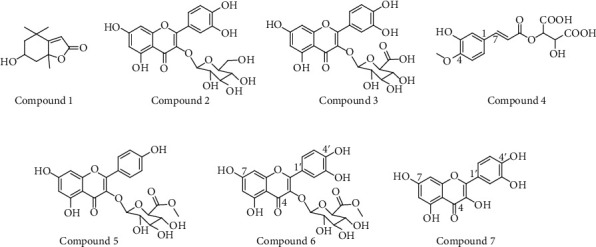
Structures of the Compounds **1**–**7** from aerial parts of *Persicaria senticosa.*

*Persicaria senticosa* aerial parts (1.4 kg), dried in the shade and powdered, were added to 40 L of 70% ethanol (HPLC grade) and two times at room temperature (each time for 2 days) and were concentrated in vacuum at 40°C to yield 180.4 g of extracts. The extracts were suspended in distilled water and then partitioned with n-hexane (4.0 L×3), CH_2_Cl_2_ (4.0 L × 3), EtOAc (4.0 L × 3), and n-butanol (4.0 L × 3) to give n-hexane (11.7 g), CH_2_Cl_2_ (7.4 g), EtOAc (21.7 g), n-butanol (22.5 g), and water-soluble fractions (110.1 g), (Scheme 1). The fraction of EtOAc (21.7 g) was separated by MPLC that used gradient mixtures as eluents (F001-003). Compounds **1** (3.2 mg) and **5** (3.9 mg) were isolated from F002 that used by prep-HPLC. The fraction F001 was separated by MPLC that used gradient mixtures as eluents (F011-F018). Compound **6** (1.5 mg) was isolated from F016 that was used by preparative HPLC. Compound **7** (2.7 mg) was isolated from F017 that was used by preparative HPLC. F012 was separated by using MPLC which used gradient mixtures as eluents (F121-F124). Compound **4** (10.8 mg) was isolated from F124 that used by preparative HPLC. In addition, F014 was separated by preparative HPLC using gradient mixtures as eluents (F141-F143). Compound **3** (2.3 mg) was isolated from F141 using preparative HPLC. Compound **2** (4.8 mg) was isolated from F142 using semipreparative HPLC. The purification of the EtOAc soluble layer was performed by column chromatography separation and MPLC analysis to Compounds **1**-**7**. It was identified as loliolide (**1**) [14], quercetin-3-O-glucoside (**2**) [15], quercetin-3-O-glucuronide (**3**) [16], 4-methoxy caftraric acid (**4**) [17], kaempferol-3-(6-methylglucuronide) (**5**) [18], quercetin-3-(6-methylglucuronide) (**6**) [19, 20], and quercetin (**7**) [21]. Structure was elucidated by a combination of 1D and 2D NMR and MS spectrometry as well as comparison with reported literatures (Figure 1).

### 3.4. Skin-Related Properties of Compounds from *Persicaria senticosa*

Radical scavenging effect on DPPH, tyrosinase inhibition, and nitric oxide assay on several compounds from *Persicaria senticosa* was found to show potent activity (Fig. S4–7). The results showed that Compound **7** has the NO assay (IC_50_ 29.7 *μ*g/mL). For DPPH, the IC_50_ of Compounds **2**, **3**, **5**, and **7** was 39.6, 31.2, 37.0, and 22.7 *μ*g/mL, respectively. In tyrosinase inhibitory activity, the IC_50_ of Compound **7** was 14.3 *μ*g/mL (Table 3).

## 4. Conclusions

*Persicaria senticosa* is an annual plant in the family Polygonaseae which is distributed in whole Korea. Since early times, this plant has been used as folk medicine with beneficial effects for the treatment of various diseases. This study evaluated skin-related properties and constituents from the aerial part extract of *Persicaria senticosa* and thirty-four Polygonaseae plants. In the course of screening for cosmetic ingredients by measuring the radical scavenging effect on DPPH, ABTS radical scavenging, antiwrinkle was evaluated using elastase inhibition, whitening was studied by tyrosinase inhibition, and anti-inflammatory was tested on nitric oxide assay. Several Polygonum extracts were found to show potent activity. The results showed that the *Persicaria senticosa* methanolic extract has the DPPH and ABTS radical scavenging activities (IC_50_ 61.0 and 17.5 *μ*g/mL). In the elastase inhibition assay and nitric oxide assay, the IC_50_ of methanolic extract of *Persicaria senticosa* was 241.5 *μ*g/mL and 71.8 *μ*g/mL, respectively. The *Persicaria senticosa* 70% ethanolic extract was partitioned with n-hexane, CH_2_Cl_2_, EtOAc, n-BuOH, and aqueous fractions.

**Figure 2:**
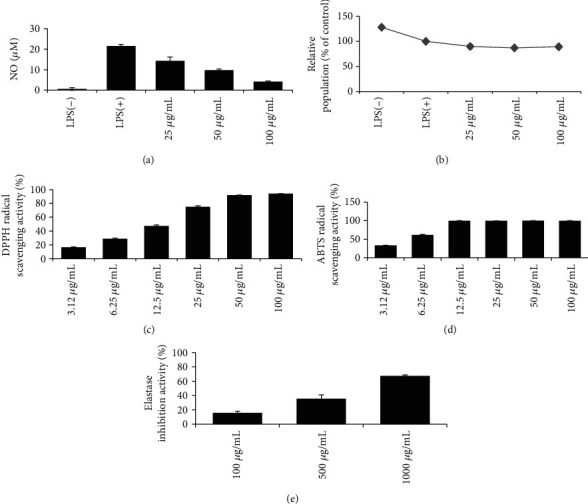
Skin-related activity of EtOAc soluble fraction of Persicaria senticosa. A ::(a) nNitric oxide assay, . B ::(b) MTT assay, . C ::(c) DPPH radical scavenging, . D ::(d) ABTS radical scavenging, . E ::(e) eElastase inhibition activity.

EtOAc soluble fraction showed a potent skin-related activity (Figure 2 and Table 3). These results suggested that active components in *Persicaria senticosa* responsible for the skin-related activity were concentrated in the EtOAc soluble fraction of *Persicaria senticosa* (Scheme 1). On the other hand, the n-hexane, dichloromethane soluble fraction, and the BuOH soluble fraction derived from the extract of *Persicaria senticosa* showed equipotent activity on nitric oxide assay, DPPH, and ABTS radical scavenging activity, whereas remaining water fraction demonstrated poor inhibitory effects (Table 2).

Thus, bioassay-guided purification of three active fractions, i.e., the EtOAc soluble fraction of *Persicaria senticosa* was conducted to purify the active principles responsible for the skin-related activity followed by the process described in Scheme 1, respectively.

**Figure 3:**
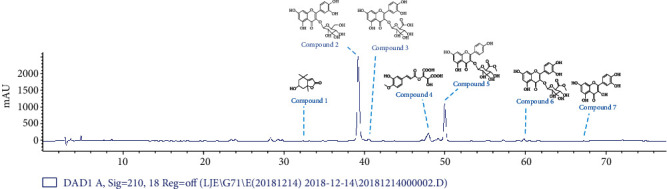
HPLC profile of EtOAc fraction of *Persicaria senticosa.*

**Figure 4:**
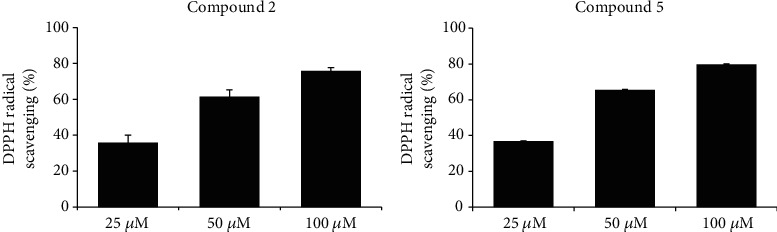
DPPH radical scavenging activity of compounds 2 and 5 from EtOAc soluble fraction of *Persicaria senticosa.*

The purification of the EtOAc soluble layer from *Persicaria senticosa* 70% ethanolic extract was performed by column chromatography separation and MPLC analysis to Compounds **1**-**7**. It was identified as loliolide (**1**), quercetin-3-O-glucoside (**2**), quercetin-3-O-glucuronide (**3**), 4-methoxy caftraric acid (**4**), kaempferol-3-(6-methylglucuronide) (**5**), quercetin-3-(6-methylglucuronide) (**6**), and quercetin (**7**). Structure was elucidated by a combination of 1D and 2D NMR and MS spectrometry as well as comparison with reported literatures (Fig. S2). Radical scavenging effect on 1,1-diphenyl-2-picrylhydrazyl(DPPH), tyrosinase inhibition, and nitric oxide assay on several compounds from *Persicaria senticosa* was found to show potent activity. The results showed that Compound **7** has the NO assay (IC_50_ 29.7 *μ*M). For DPPH, the IC_50_ of Compounds **2**, **3**, **5**, and **7** was 39.4, 32.1, 37.0, and 22.7 *μ*M, respectively. In tyrosinase inhibitory activity, the IC_50_ of Compound **7** was 14.3 *μ*M. As shown in Figure 3, Compounds **2** and **5** are the main compounds in the EtOAc soluble fraction of *Persicaria senticosa*(Fig. S1). And Compounds **2** and **5** showed excellent antioxidant activity(Figure 4), which is consistent with previous studies [18, 22].

This present study demonstrated that *Persicaria senticosa* and Polygonaseae contain chemical compounds with good skin-related activities and could be interesting as a novel source of bioactive agents for cosmetic industries.

## Figures and Tables

**Table 1:** Skin-related properties of plants extracts in the family Polygonaseae.

No.	Name	Parts	Anti-inflammatory		Antioxidant		Whitening	Anti-wrinkle
			NO (*μ*g/mL)	MTT (*μ*g/mL)	DPPH (*μ*g/mL)	ABTS (*μ*g/mL)	Tyrosinase assay (*μ*g/mL)	Elastase assay (*μ*g/mL)
1	*Polygonum sachalinense*	Fruits	71.1 ± 6.9	>100	98.5 ± 2.4	6.0 ± 0.1	308.8 ± 9.2	<100
		Root	>100	>100	54.3 ± 1.7	9.3 ± 0.2	289.0 ± 6.1	<100
2	*Rumex japonicus*	Whole plants	>100	>100	>100	31.1 ± 0.3	>1000	400.6 ± 4.3
3	*Polygonum ciliinerve*	Whole plants	>100	>100	36.9 ± 1.2	6.6 ± 0.1	>1000	216.0 ± 5.0
4	*Polygonum manshuriense*	Whole plants	>100	>100	86.3 ± 1.5	23.5 ± 0.1	>1000	564.0 ± 9.3
5	*Polygonum cuspidatum*	Whole plants	>100	>100	43.3 ± 0.9	6.8 ± 0.0	>1000	<100
6	*Persicaria hydropiper*	Whole plants	83.2 ± 3.6	>100	35.1 ± 1.1	10.5 ± 0.3	>1000	<100
**7**	*Polygonum senticosum*	Whole plants	>100	>100	91.7 ± 0.3	25.7 ± 0.2	>1000	263.9 ± 3.8
		Aerial parts	71.8 ± 3.7	>100	61.0 ± 0.6	17.5 ± 0.1	>1000	739.7 ± 6.5
8	*Persicaria sieboldi*	Whole plants	>100	>100	>100	26.8 ± 0.2	>1000	<100
9	*Polygonum orientale*	Fruits	54.5 ± 2.3	>100	50.6 ± 3.3	14.4 ± 0.1	445.4 ± 5.3	<100
		Whole plants	73.5 ± 5.2	>100	30.8 ± 0.2	8.8 ± 0.1	391.7 ± 2.3	<100
10	*Polygonum cuspidata*	Seed	64.8 ± 1.9	>100	24.0 ± 0.7	5.2 ± 0.1	483.9 ± 4.2	189.5 ± 7.3
11	*Persicaria tinctoria*	Flora	>100	>100	44.8 ± 0.4	11.6 ± 0.1	>1000	129.5 ± 7.5
12	*Rumex conglomeratus*	Aerial parts	>100	>100	48.2 ± 1.6	18.9 ± 0.2	>1000	536.9 ± 2.9
		Root	>100	>100	37.2 ± 0.3	11.6 ± 0.1	>1000	348.0 ± 7.3
13	*Persicaria lapathifolia*	Whole plants	>100	>100	29.3 ± 0.5	10.4 ± 0.1	>1000	<100
14	*Persicaria dissitiflora*	Whole plants	>100	>100	30.0 ± 1.0	10.8 ± 0.1	411.2 ± 4.8	<100
15	*Persicaria thunbergii*	Aerial parts	65.7 ± 12.3	>100	>100	49.4 ± 0.2	>1000	>1000
		Whole plants	60.3 ± 1.3	>100	>100	40.2 ± 0.2	>1000	>1000
16	*Polygonum aviculare*	Whole plants	74.5 ± 4.2	>100	22.6 ± 0.3	9.3 ± 0.1	>1000	133.3 ± 3.2
17	*Polygonum emaginatum*	Whole plants	79.3 ± 1.3	>100	>100	20.4 ± 0.1	>1000	832.9 ± 9.8
18	*Rumex obtusifolius*	Aerial parts	>100	>100	>100	59.5 ± 0.8	>1000	>1000
		Root	>100	>100	42.0 ± 0.1	14.1 ± 0.1	>1000	348.8 ± 4.7
19	*Rumex acetosella*	Whole plants	>100	>100	34.2 ± 0.5	9.4 ± 0.2	733.1 ± 16.5	<100
		Whole plants	>100	>100	45.0 ± 0.5	11.9 ± 0.1	876.2 ± 7.9	<100
20	*Rumex crispus*	Aerial parts	90.4 ± 4.3	>100	30.5 ± 0.3	10.8 ± 0.2	>1000	<100
		Root	>100	>100	71.1 ± 0.1	22.7 ± 0.4	>1000	438.1 ± 9.8
		Whole plants	>100	>100	39.1 ± 1.1	15.4 ± 0.4	>1000	318.1 ± 7.6
21	*Persicaria nepalensis*	Whole plants	90.8 ± 3.1	>100	50.3 ± 0.2	17.2 ± 0.1	>1000	>1000
22	*Polygonum alpinum*	Aerial parts	83.3 ± 6.8	>100	28.3 ± 0.3	9.8 ± 0.1	>1000	<100
		Aerial parts	71.6 ± 7.9	>100	15.4 ± 0.5	5.3 ± 0.1	564.8 ± 4.7	<100
		Whole plants	72.1 ± 5.3	>100	22.2 ± 0.1	9.0 ± 0.2	>1000	<100
23	*Persicaria longiseta*	Whole plants	>100	>100	44.9 ± 0.4	12.2 ± 0.1	>1000	<100
24	*Persicaria chinensis*	Whole plants	72.1 ± 9.3	>100	19.2 ± 0.5	7.2 ± 0.0	726.4 ± 6.9	<100
25	*Persicaria japonica*	Whole plants	45.3 ± 5.5	>100	24.4 ± 0.7	9.4 ± 0.1	431.1 ± 7.8	<100
		Leaf, stem	>100	>100	26.4 ± 1.0	10.7 ± 0.3	>1000	<100
26	*Persicaria viscofera*	Whole plants	86.5 ± 2.7	>100	39.1 ± 0.6	11.4 ± 0.0	694.9 ± 6.8	<100
27	*Rumex longifolius*	Aerial parts	<25	>100	>100	78.2 ± 0.2	>1000	>1000
		Aerial parts	82.4 ± 5.8	>100	>100	82.0 ± 1.1	>1000	>1000
28	*Persicaria conspicua*	Whole plants	>100	>100	48.1 ± 0.6	16.8 ± 0.2	>1000	<100
29	*Persicaria sieboldi*	Whole plants	>100	>100	80.3 ± 0.8	21.9 ± 0.4	>1000	406.2 ± 7.4
30	*Persicaria perfoliata*	Aerial parts	52.2 ± 6.4	>100	68.8 ± 0.5	17.4 ± 0.4	>1000	352.1 ± 3.2
		Whole plants	>100	>100	50.4 ± 2.4	15.4 ± 0.1	>1000	164.0 ± 4.1
31	*Rheum palmatum*	Whole plants	71.8 ± 6.8	>100	27.3 ± 0.8	8.6 ± 0.2	>1000	<100
32	*Rumex acetosa*	Whole plants	>100	>100	>100	59.8 ± 1.2	>1000	>1000
33	*Polygonum multiflorum*	Whole plants	>100	>100	51.7 ± 0.9	16.1 ± 0.1	>1000	647.4 ± 7.4
34	*Persicaria lapathifolia*	Whole plants	86.9 ± 5.2	>100	24.5 ± 0.8	11.1 ± 0.1	>1000	<100
P.C.			L-NMMA 36.3 ± 6.1 *μ*M	L-NMMA >100 *μ*M	BHA 15.6 ± 0.2	BHA 2.9 ± 0.1	Arbutin 135.0 ± 19.9	Ursolic acid 53.2 ± 6.1

**Table 2:** Skin-related properties of *Persicaria senticosa* fractions.

Fractions	Anti-inflammatory		Antioxidant		Antiwrinkle
	NO (*μ*g/mL)	MTT (*μ*g/mL)	DPPH (*μ*g/mL)	ABTS (*μ*g/mL)	Elastase assay
*Persicaria senticosum Hx fr.*	57.72 ± 5.54	>100	43.48 ± 1.20	22.71 ± 0.46	<100
*Persicaria senticosum CH_2_Cl_2_ fr.*	<25	>100	20.19 ± 0.39	7.88 ± 0.24	<100
*Persicaria senticosum EtOAc fr.*	44.64 ± 7.86	>100	13.69 ± 0.74	4.97 ± 0.09	713.56 ± 36.44
*Persicaria senticosum BuOH fr.*	32.66 ± 3.95	>100	33.87 ± 1.36	11.51 ± 0.25	123.55 ± 87.76
*Persicaria senticosum H_2_O fr.*	>100	>100	>100	>100	>1000
P. C.	L-NMMA 37.63 ± 6.10 *μ*M	L-NMMA >100 *μ*M	BHA 14.58 ± 0.06	BHA 4.03 ± 0.05	Ursolic acid 38.97 ± 7.12

**Table 3:** Skin-related properties of compounds from *Persicaria senticosa.*

Sample	NO assay IC_50_ (*μ*M)	MTT assay IC_50_ (*μ*M)	DPPH assay IC_50_ (*μ*M)	Elastase inhibition assay IC_50_ (*μ*M)	Tyrosinase inhibition assay IC_50_ (*μ*M)
Comp. **1**	>100	>100	>1000	>1000	>100
Comp. **2**	>100	>100	39.56 ± 4.10	>1000	>100
Comp. **3**	>100	>100	31.15 ± 0.47	>1000	>100
Comp. **4**	>100	84.56 ± 9.85	>1000	>1000	>100
Comp. **5**	>100	>100	36.99 ± 0.16	>1000	>100
Comp. **6**	>100	>100	>1000	>1000	>100
Comp. **7**	29.69 ± 7.07	>100	22.71 ± 0.53	>1000	14.31 ± 3.93
P. C.	L-NMMA 14.56 ± 3.46	L-NMMA >100	BHA 271.70 ± 19.45	Ursolic acid 86.3 ± 9.90	Kojic acid 11.38 ± 4.16

## Data Availability

The data used to support the findings of this study are included in the Supplemental Information File.

## Supplementary Materials

Supplementary Materials Attached is the NMR as supplementary data for this research work (Supplementary Materials). Figure S1: specimen photo of *Persicaria senticosum.* Figure S2: DPPH radical scavenging activity assay data of isolated compounds from *Persicaria senticosum.* Figure S3: tyrosinase inhibition assay data of isolated compounds from *Persicaria senticosum*. Figure S4: NO assay data of isolated compounds from *Persicaria senticosum.* Figure S5: cell cytotoxicity assay data of isolated compounds from *Persicaria senticosum.* Figure S6: spectral data of isolated compounds *Persicaria senticosum* (Compounds 1–7). Figure S7: high-performance liquid chromatography (HPLC) condition and profile of EtOAc fraction of *Persicaria senticosum*.
