# Supplementary material for: Skin-Related Properties and Constituents from the Aerial Parts Extract of Persicaria senticosa
Source: Oxid Med Cell Longev. 2020 Dec 19;2020:6627752. doi: 10.1155/2020/6627752 (PMC7769672; doi:10.1155/2020/6627752)

**
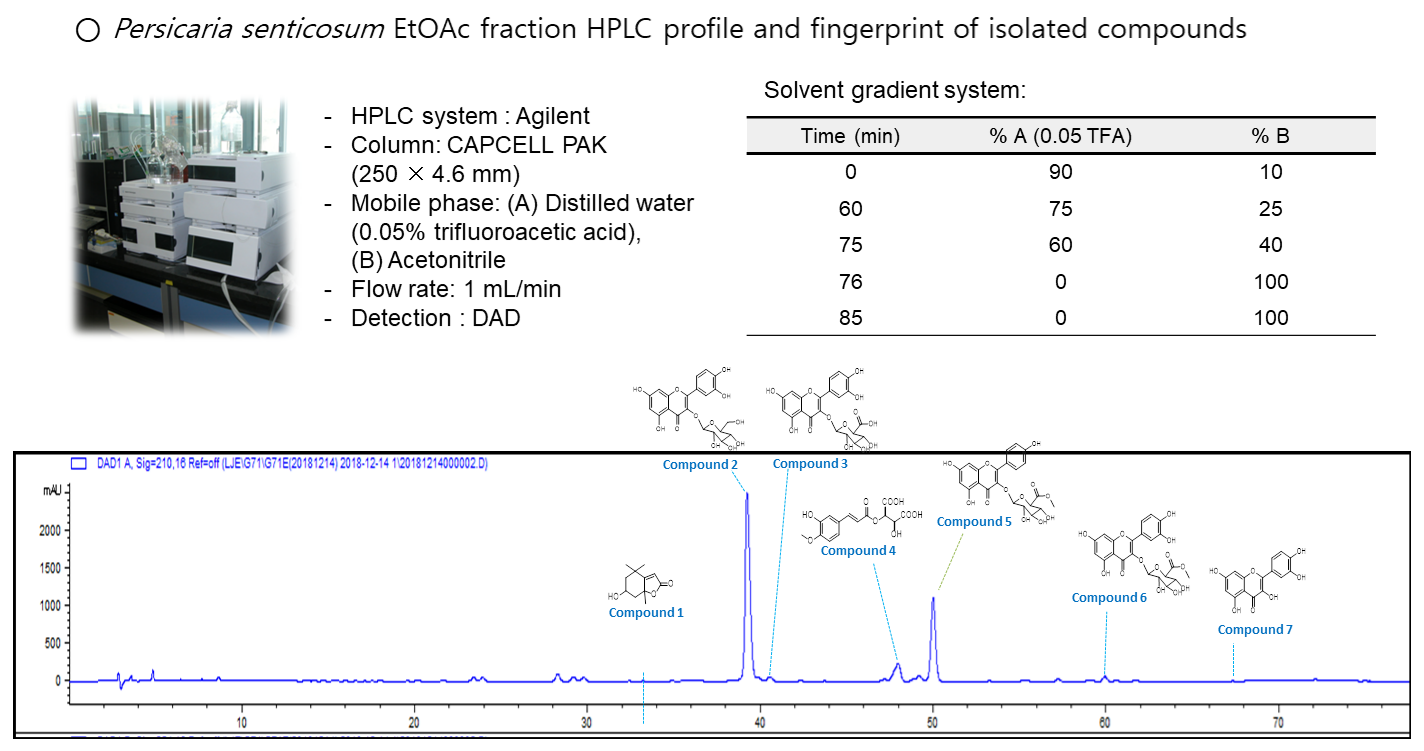
Supplementary materials**

**Figure S1. High-performance liquid chromatography (HPLC) analysis of *Persicaria senticosum.***


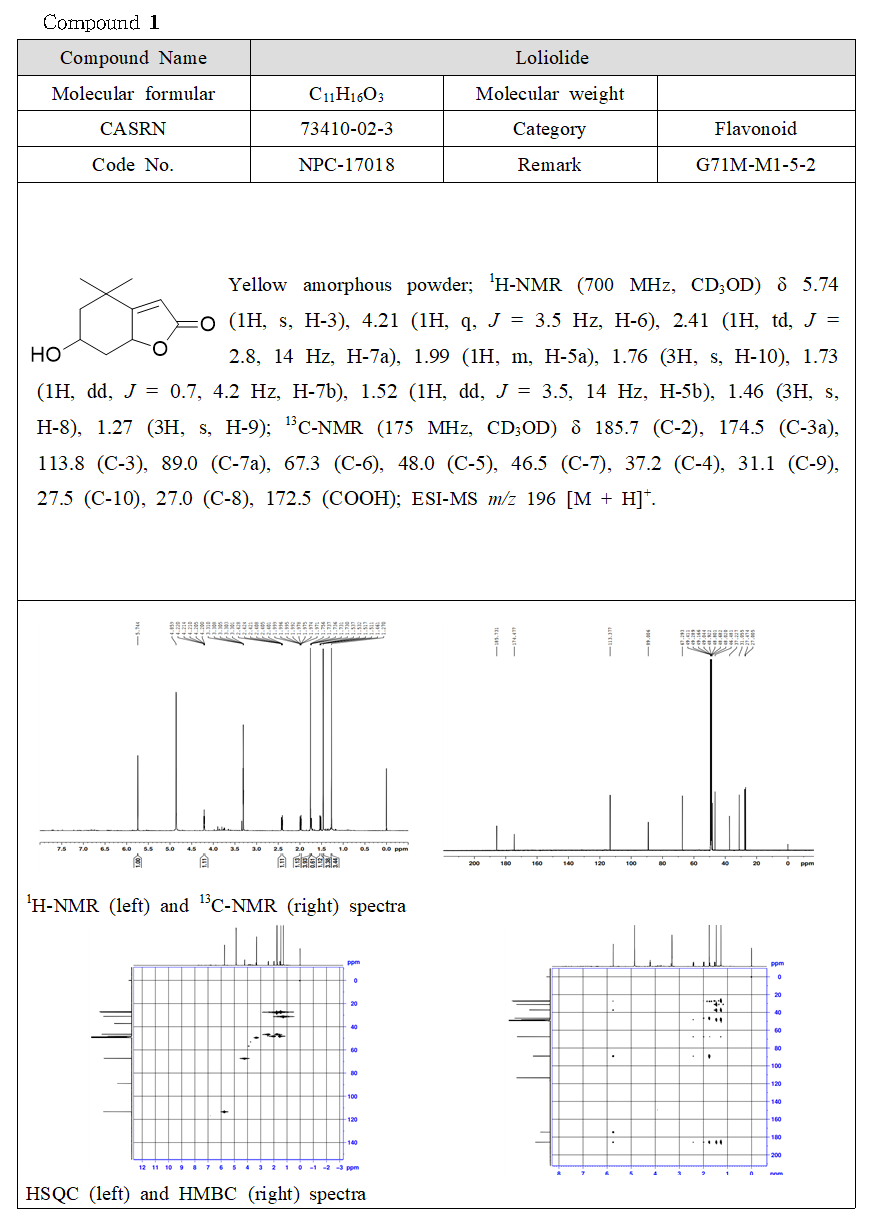
**Figure S2. Spectral data of isolated compounds *Persicaria senticosum.* (Compound 1 - 7)**


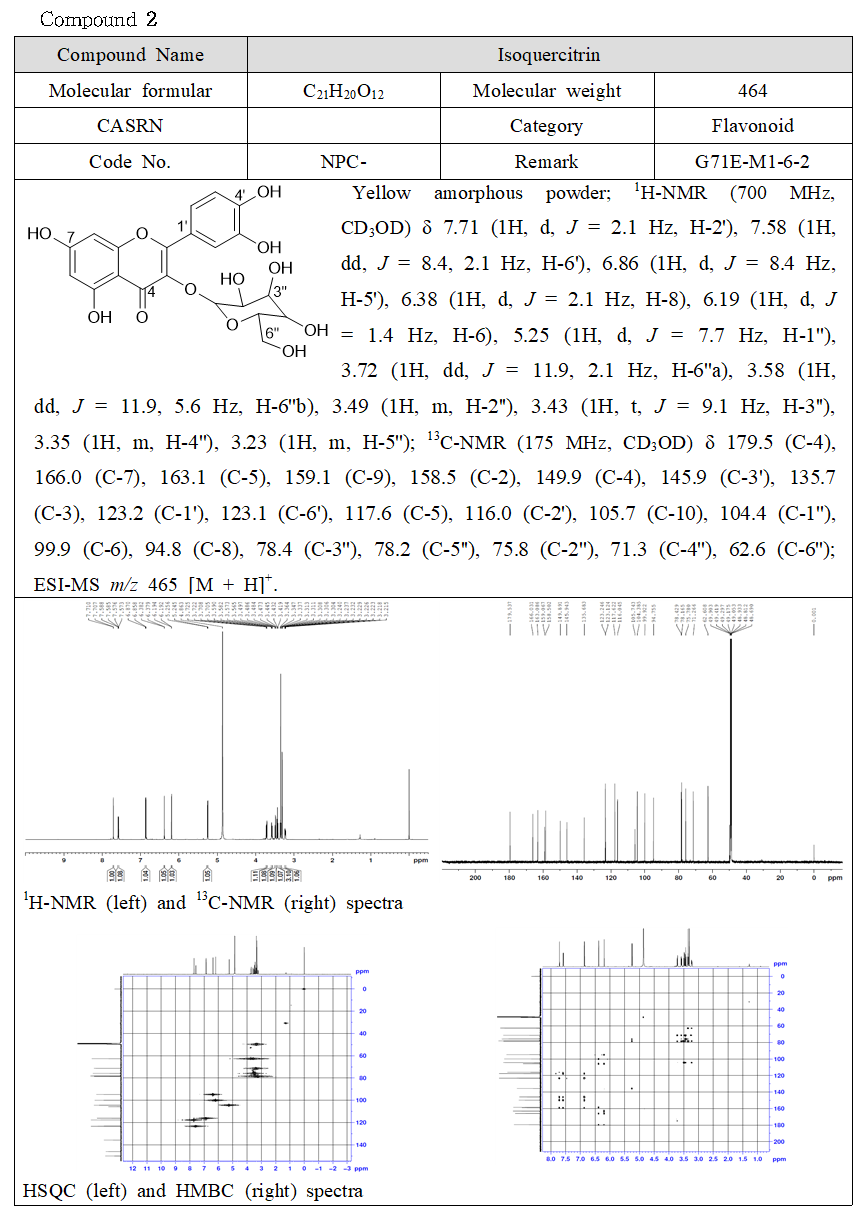


**
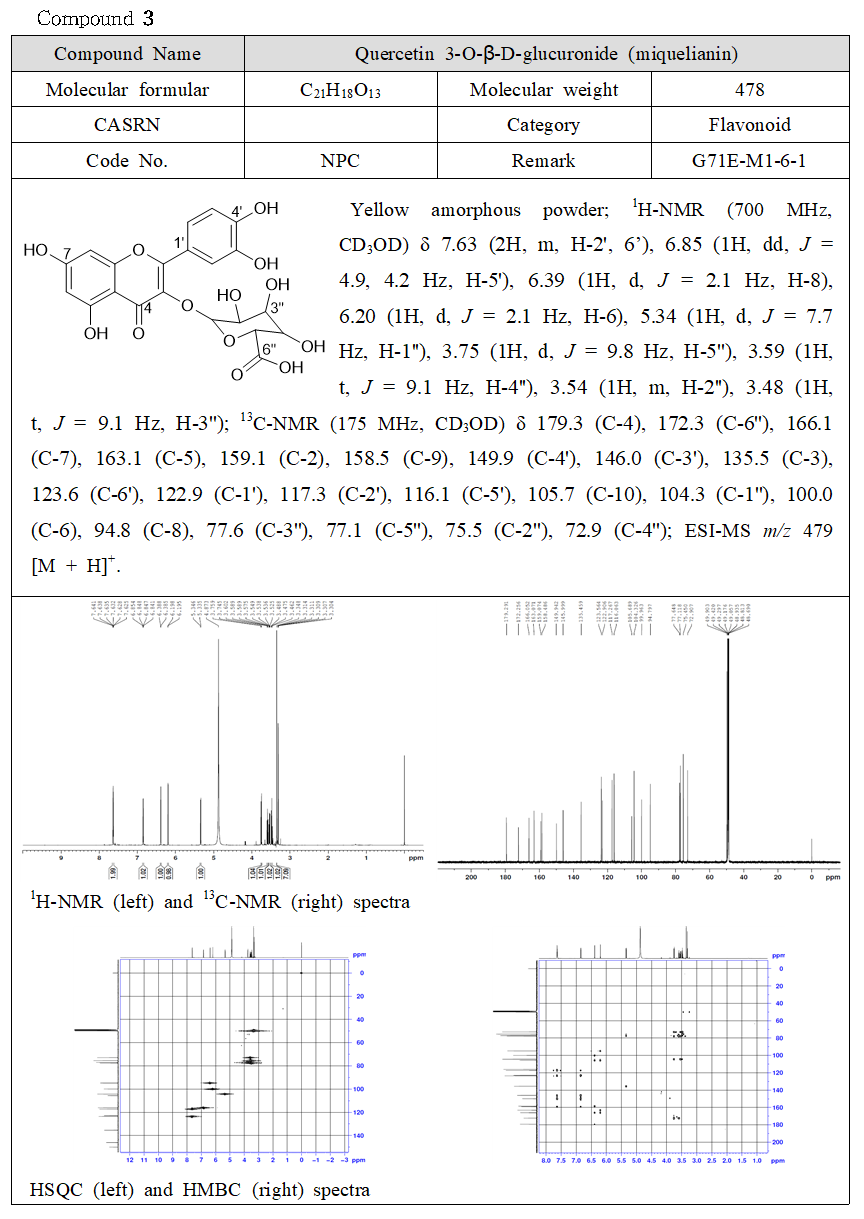
**

**
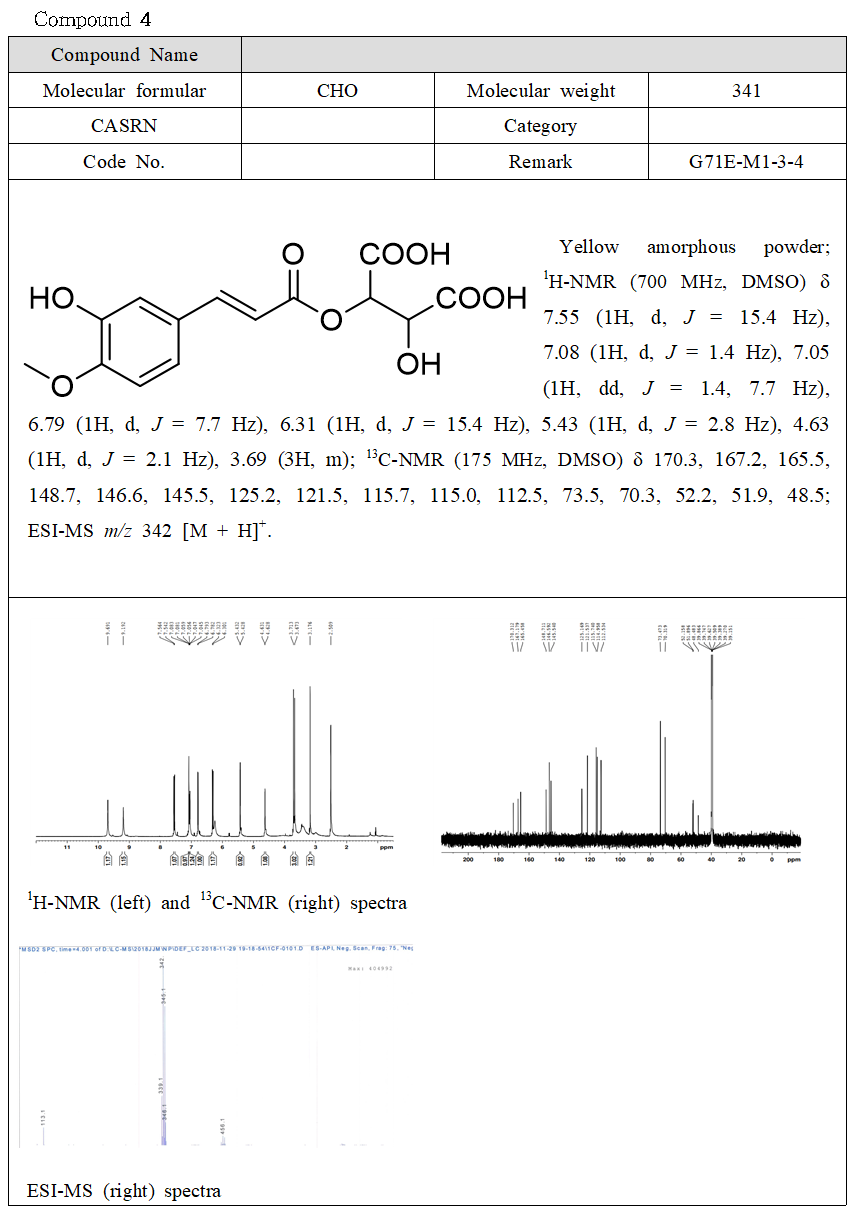
**

**
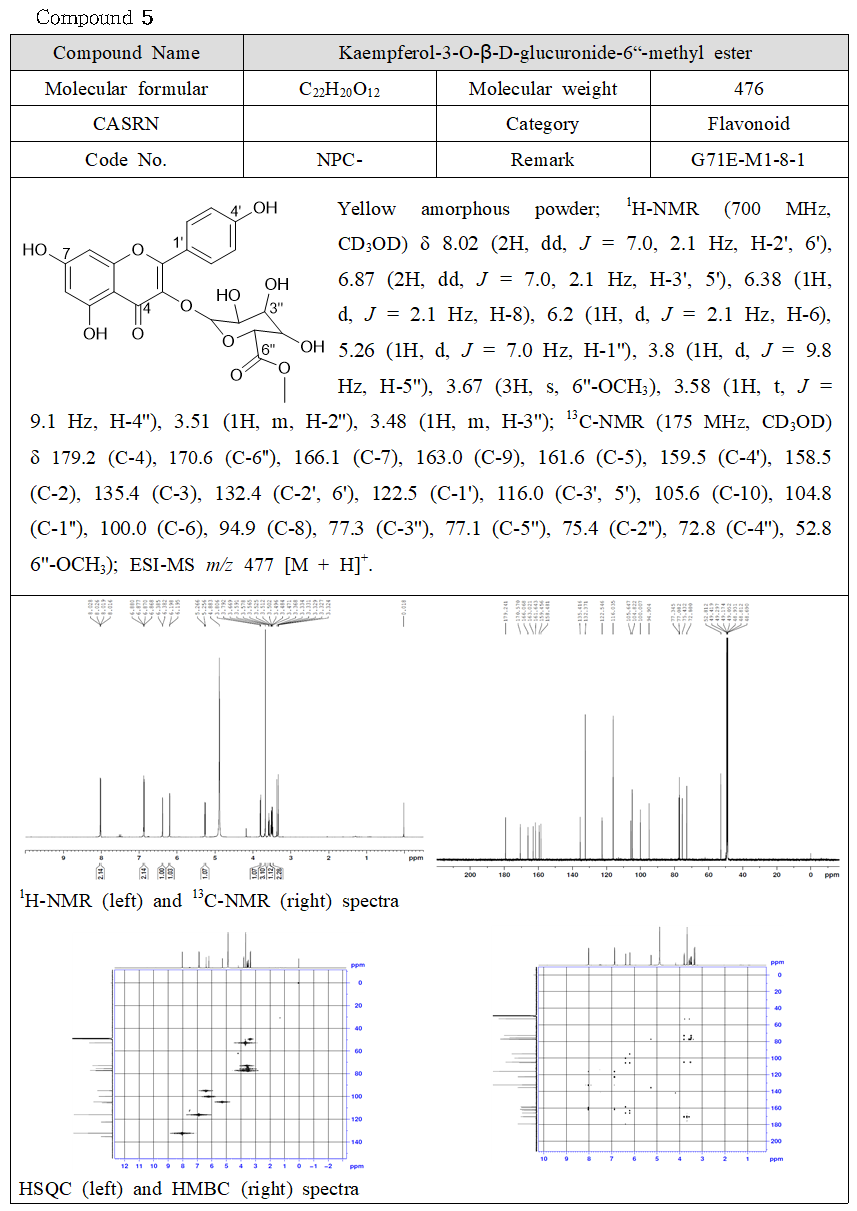
**

**
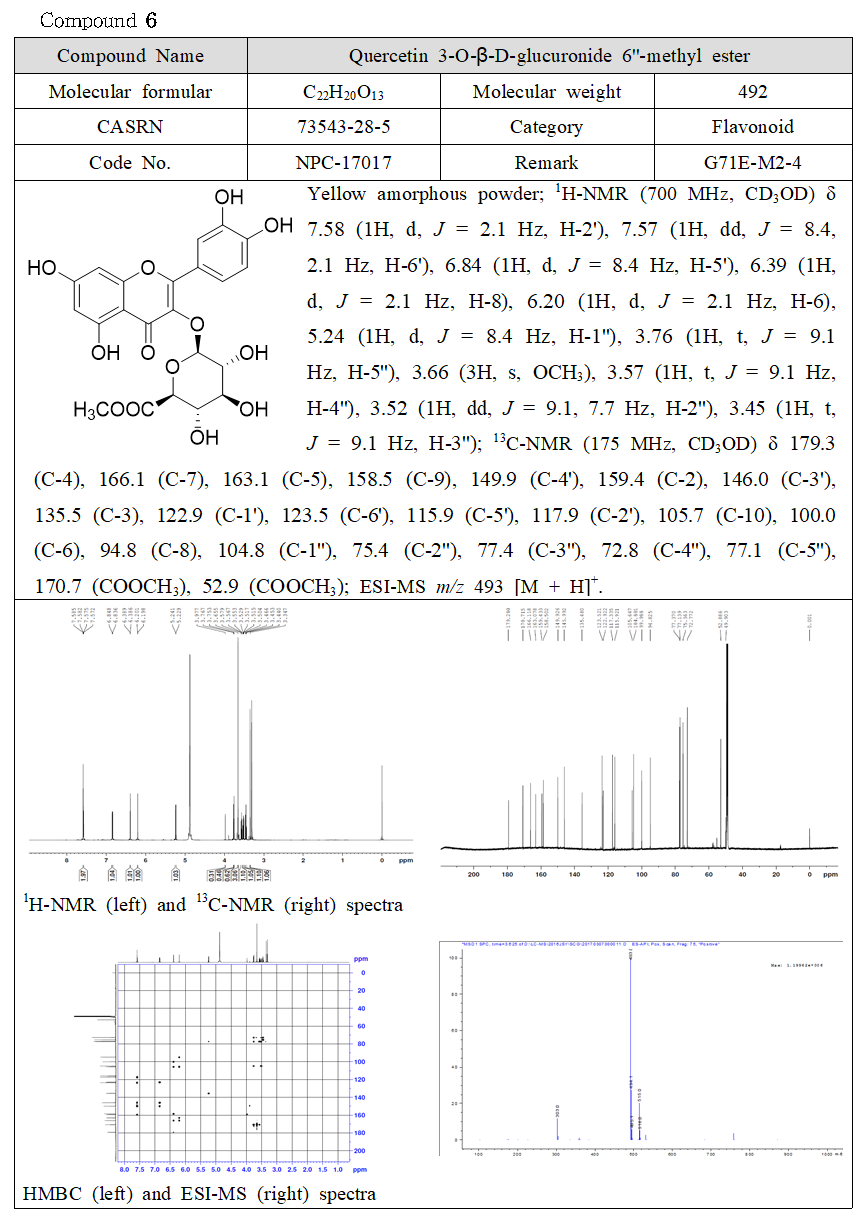
**

**
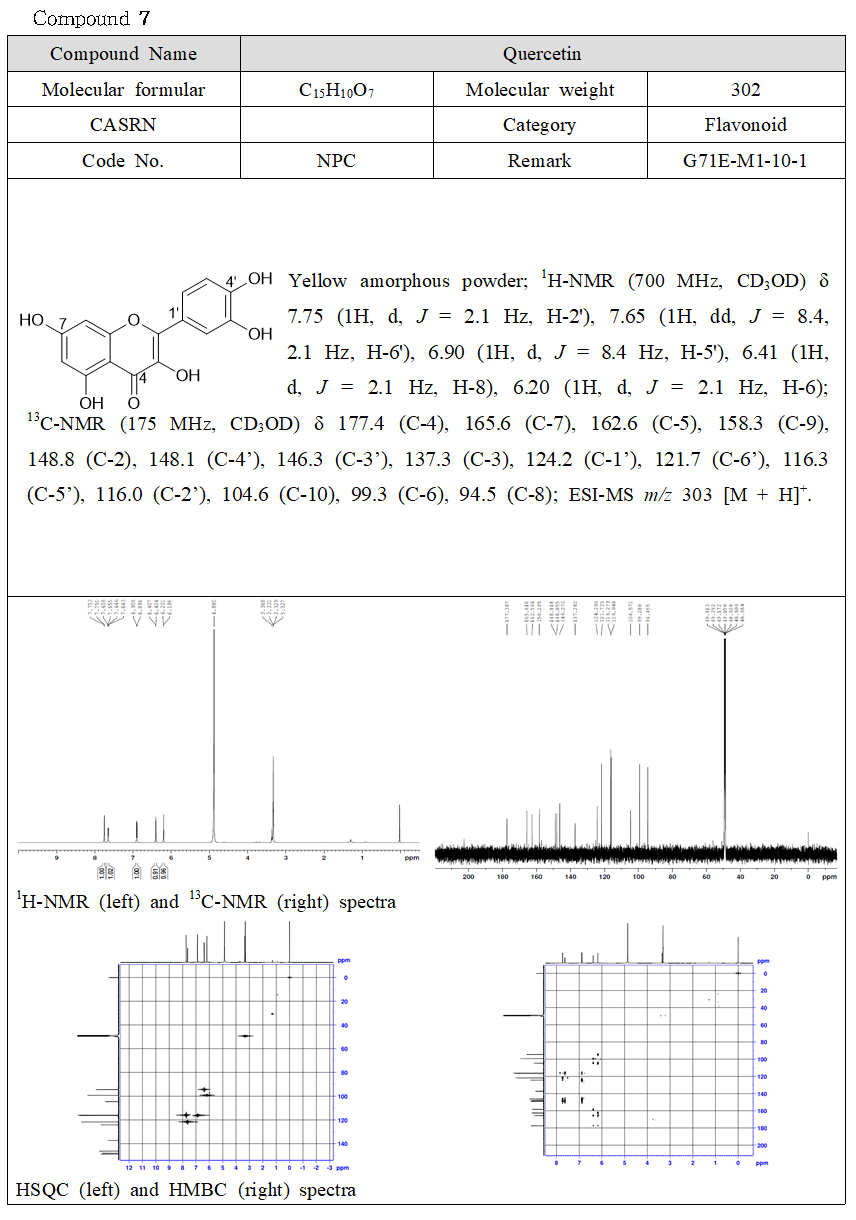
**

**Figure S3. Specimen photo of *Persicaria senticosum.***

**
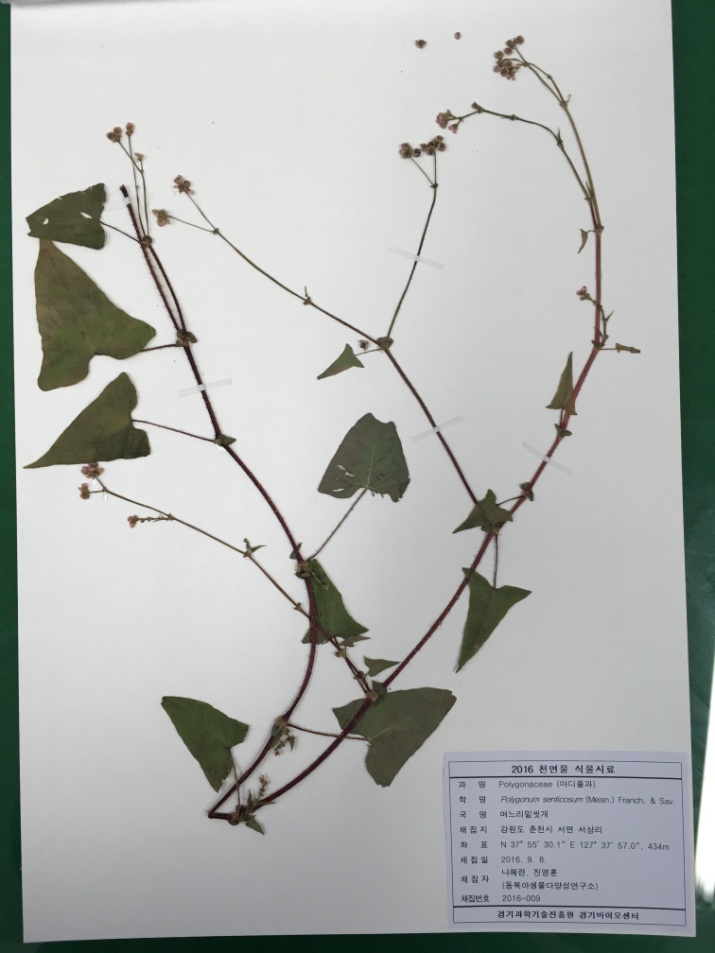
**

**Figure S4. DPPH radical scavenging activity assay data of isolated compounds from *Persicaria senticosum.***

**
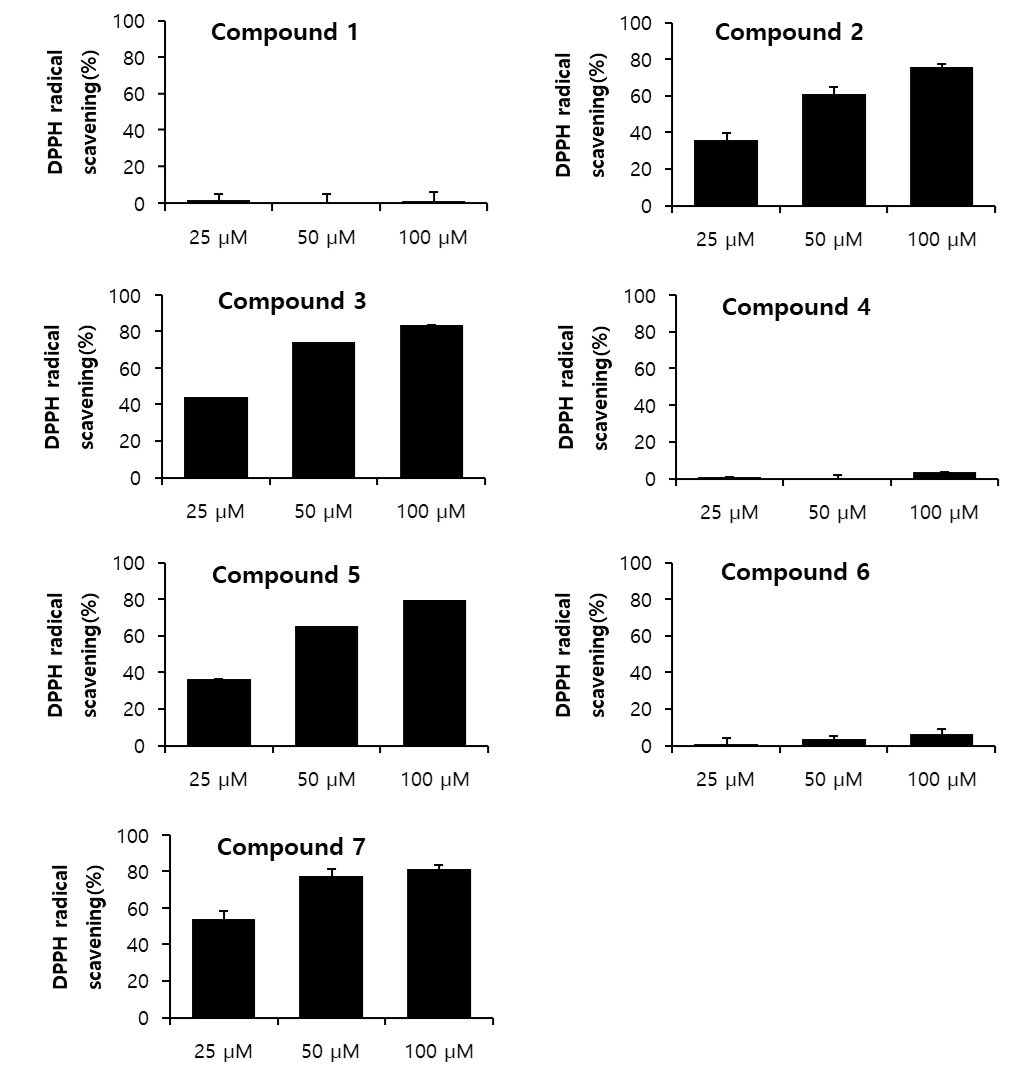
**

**Figure S5.** **Tyrosinase inhibition assay data of isolated compounds from *Persicaria senticosum.***

**
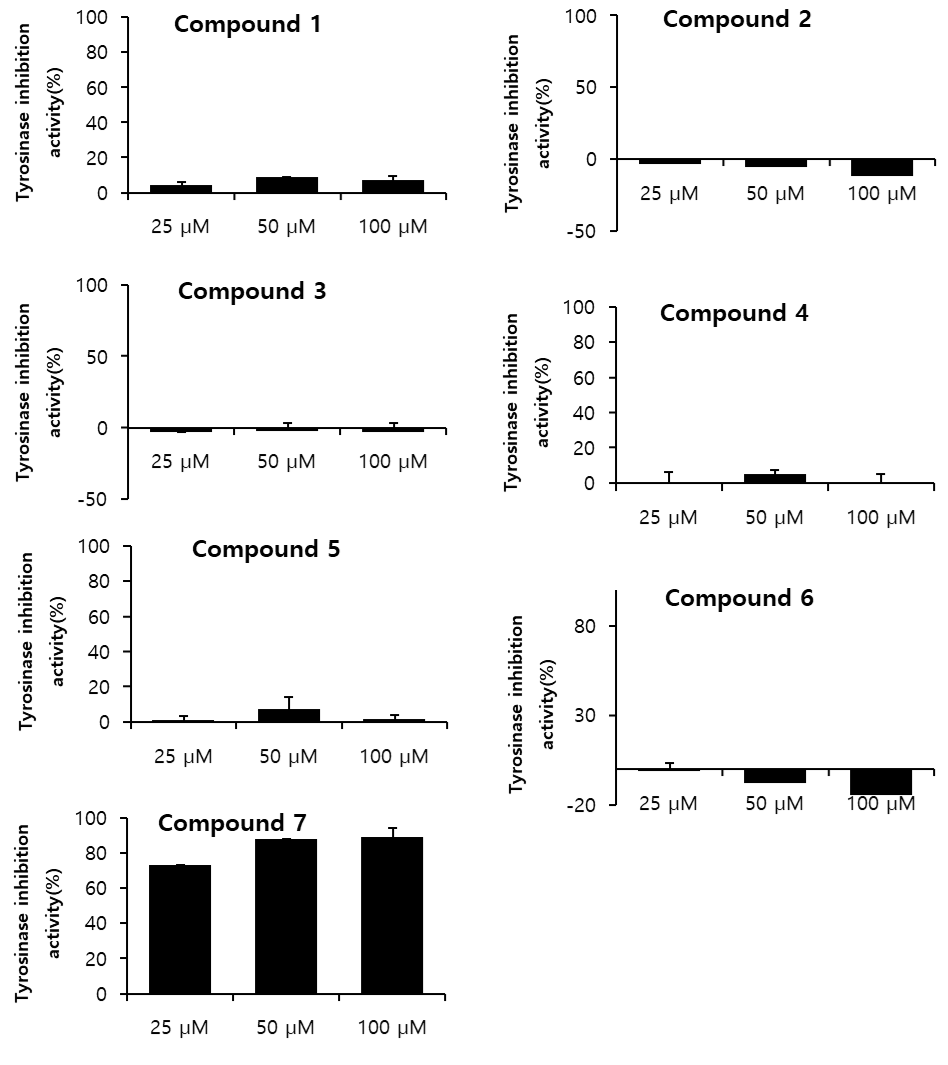
**

**Figure S6.** **NO assay data of isolated compounds from** ***Persicaria senticosum.***


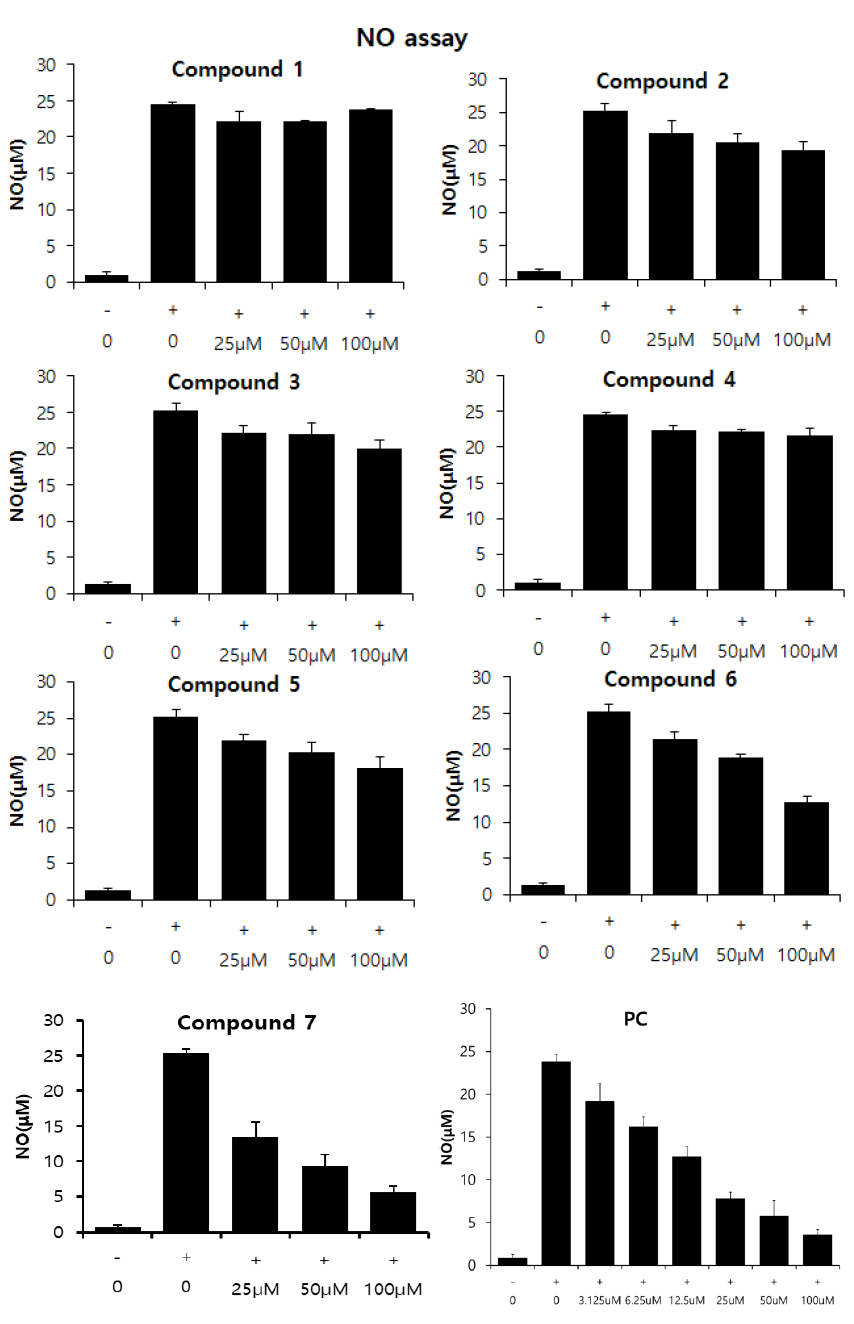


**Figure S7.** **Cell cytotoxicity assay data of isolated compounds from** ***Persicaria senticosum.***


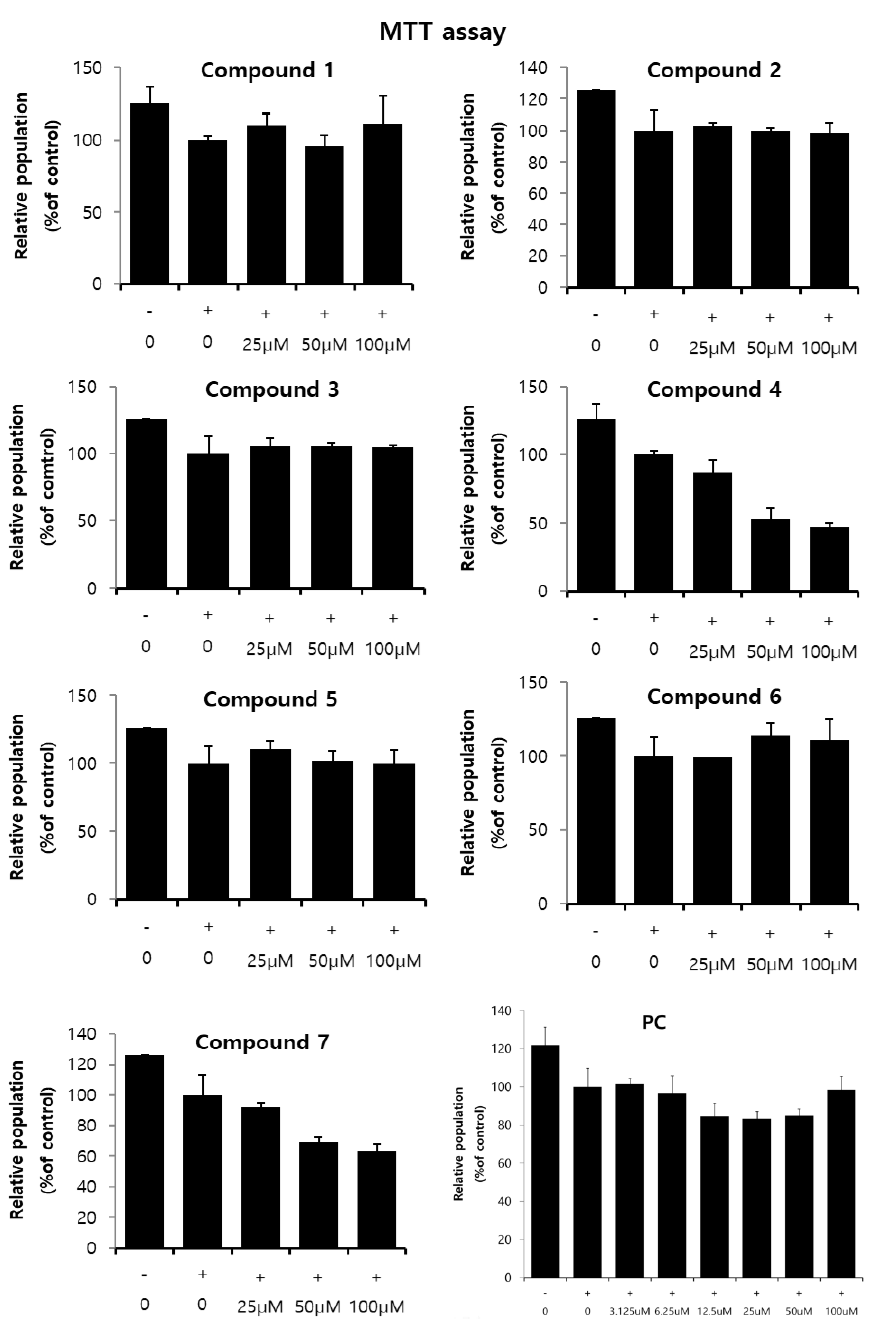

Supplement: Supplementary Materials — Attached is the NMR as supplementary data for this research work (Supplementary Materials). Figure S1: specimen photo of Persicaria senticosum. Figure S2: DPPH radical scavenging activity assay data of isolated compounds from Persicaria senticosum. Figure S3: tyrosinase inhibition assay data of isolated compounds from Persicaria senticosum. Figure S4: NO assay data of isolated compounds from Persicaria senticosum. Figure S5: cell cytotoxicity assay data of isolated compounds from Persicaria senticosum. Figure S6: spectral data of isolated compounds Persicaria senticosum (Compounds 1–7). Figure S7: high-performance liquid chromatography (HPLC) condition and profile of EtOAc fraction of Persicaria senticosum. [file 6627752.f1.docx]
